# EEG Signal and Feature Interaction Modeling-Based Eye Behavior Prediction Research

**DOI:** 10.1155/2020/2801015

**Published:** 2020-05-16

**Authors:** Pengcheng Ma, Qian Gao

**Affiliations:** School of Computer Science and Technology, Qilu University of Technology (Shandong Academy of Sciences), Jinan Shandong 250353, China

## Abstract

In recent years, with the development of brain science and biomedical engineering, as well as the rapid development of electroencephalogram (EEG) signal analysis methods, using EEG signals to monitor human health has become a very popular research field. The innovation of this paper is to analyze the EEG signal for the first time by building a depth factorization machine model, so that on the basis of analyzing the characteristics of user interaction, we can use EEG data to predict the binomial state of eyes (open eyes and closed eyes). The significance of the research is that we can diagnose the fatigue and the health of the human body by detecting the state of eyes for a long time. On the basis of this inference, the proposed method can make a further useful auxiliary support for improving the accuracy of the recommendation system recommendation results. In this paper, we first extract the features of EEG data by wavelet transform technology and then build a depth factorization machine model (FM+LSTM) which combines factorization machine (FM) and Long Short-Term Memory (LSTM) in parallel. Through the test of real data set, the proposed model gets more efficient prediction results than other classifier models. In addition, the model proposed in this paper is suitable not only for the determination of eye features but also for the acquisition of interactive features (user fatigue) in the recommendation system. The conclusion obtained in this paper will be an important factor in the determination of user preferences in the recommendation system, which will be used in the analysis of interactive features by the graph neural network in the future work.

## 1. Introduction

The electrical activity of the cerebral cortex is recorded by detecting electrodes, and the potential amplitude is taken as the vertical axis and the time as the horizontal axis to form a map that can reflect the spontaneous and rhythmic electrical activity of different parts of the brain with time, which is called EEG [1]; its full name is electroencephalogram. In 1924, Hans Bergen, a German psychiatrist, first discovered and recorded the regular electrical activity of the human brain [2]. In 1933, Berger's research was affirmed by the famous British physiologist E.D. Adrian. Since then, EEG has developed rapidly, and the detection methods which include multilead regular electroencephalogram (spontaneous EGG and evoked EGG), sleep EEG, dynamic EEG, video EEG, and other detection methods have occurred [3–5]. The structure of the human brain is very complex and sophisticated. From the microscopic point of view, it is mainly composed of billions of neurons. The EEG signal consists of two parts, one is the pyramidal neurons in the cortex and the other one is the postsynaptic potential difference of the vertical dendrites. The bioelectric signal of the central nervous system is used to transmit, store, and process various physiological activity information, so as to control the human behavior [6]. As an action related to EEG, the eye state (open and close eyes) can be identified by observing the characteristic changes of EEG signals.

As the key technology of brain computer interface (BCI), EEG can be applied in five stages [7]. Among them, there are three important contents for signal processing, which are the preprocessing, feature extraction, and classification of the EEG signal; all above have been widely studied [8, 9]. As early as the end of the 19th century, British physiologist Richard Caton took the lead in using a galvanometer to capture the weak current signal on the surface of the animal's cerebral cortex [10]. Through the study of the captured electrical signals of animals, the researchers found that in the state of quiet without external stimulation, the captured current signal waveform showed rhythmic oscillation. In recent years, with the development of computer science and technology, advanced technologies such as X-ray electronic computed tomography and positron emission computed tomography have been introduced in EEG research [11]. EEG is also commonly used to monitor a patient's sleep, anesthesia depth, and fatigue. This article is to monitor the state of eyes (fatigue degree) through EEG.

A large number of scholars are trying to detect the fatigue state through EEG. The intelligent transportation laboratory (University of Pennsylvania) and NHTSA [12] use a brain wave tester and head motion detector to test and analyze the EEG and eye characteristic parameters of the driver under a fatigue state and finally determine PERCLOS (ratio of cumulative time of eye closure to unit time) as the test index of the driver's physiological fatigue degree evaluation. Bergasa et al. [13] (Australia) tested several nondrivers, and based on the changes of the brain wave in a nonfatigue state, they further analyzed the characteristics of brain wave changes in five stages of nonfatigue, near fatigue, moderate fatigue, doze, and antifatigue (wake up from fatigue). Yoshihiro Takei et al. (Japan University of Technology, Zhipu) obtained the steering angle signal of drivers when turning in the process of driving simulation on the basis of the simulation experiment test. They first processed the steering angle signal with the help of Fourier transform theory and wavelet theory. Then, they determined the wavelet transform value of the steering angle signal of drivers in different mental states by using nonlinear theory (chaos theory), so as to judge the driver's fatigue level. Finally, the fast transform algorithm (FFT, fast wavelet transform) is discussed to realize the real-time evaluation of driver fatigue state. According to four kinds of typical brain waves (*δ* wave, frequency is 1-3.5 Hz; *α* wave, frequency is 7.5-12.5 Hz; *β* wave, frequency is 12.5-30 Hz; and *θ* wave, frequency is 3.5-7.5 Hz) and their changes, we can reflect people's mental state. Relevant research shows that the change of *α* wave is the best reflection of human fatigue [14]. W-Bang et al. collected four kinds of typical brain waves, obtained the corresponding brain wave entropy using the thermodynamic entropy theory, and evaluated the fatigue degree of drivers according to the change of the calculated value [15]. Yan et al. [16] conducted experimental tests with the KT98-2000A dynamic electroencephalograph. First, the driver (healthy, not taking any irritating drugs) under nonfatigue and fatigue states was collected to engage in various driving operations (starting, shifting, controlling brain waves during movement, steering…). Next, the power spectrum is obtained, and the power spectrum density estimates of various brain waves are calculated. Finally, the average power spectral density ratios of four typical brainwaves when the driver performs various driving operations such as starting, shifting, braking, and steering under nonfatigue and fatigue conditions are obtained.

Most of the research mentioned above are to judge the fatigue state of the human body by directly recognizing the characteristics of EEG. In this paper, the feature data is extracted from EEG by wavelet transform technology, and then, the eye state is predicted by a classification algorithm, compared with traditional classification algorithms, *k*-nearest neighbor algorithm [17–19], naive Bayes algorithm [20–22], artificial neural network (ANN) [23], and linear discriminant analysis [24–27]. In this paper, we build a FM+LSTM model to predict the eye state. The model is combined with the linear interaction characteristics of FM and the nonlinear interaction characteristics of LSTM. It can greatly improve the accuracy of eye state classification and prediction. On the basis of this research, we can also infer the fatigue degree of the human body by monitoring the eye state of the human body for a long time, which can help us to recommend user behavior. For example, it can help us to detect whether the user is in the state of work overload in working time or not by combining the proposed eye state classification prediction method and our previous interaction characteristic research results. Meanwhile, it can also help us to detect whether the fatigue state of users is caused by nonworking reasons in nonworking time or not. Thus, more accuracy user behavior recommendations can be got.

## 2. Related Work

The purpose of this paper is to predict the eye state (open and closed eyes) by EEG data, which is a typical binary classification problem. Because the traditional binary classification has many shortcomings in some aspects, and its performance is poor, we use the FM algorithm which is widely used in the click rate prediction of recommendation system, as well as a good binary classification algorithm. The advantages of the FM algorithm are as follows: (1) FM model can carry out reasonable parameter trajectory in very sparse data; (2) the complexity of the FM model is linear, the optimization effect is good, and it does not need to rely on a support vector like support vector machines (SVM); and (3) FM is a general model, which can be used in any case where the eigenvalue is real. Other factorization models can only be used in some cases where the input data is relatively fixed. In addition, we also use LSTM, which is a deep network model with good generalization ability and nonlinear mapping ability. Compared with the recurrent neural network, LSTM solves the gradient explosion problem; therefore, LSTM is a strong classification prediction model.

In the experimental part of this paper, after extracting the corresponding features through wavelet transform technology, one hot coding processing is carried out on these data, which will not only increase the number of input data but also generate a large number of sparse data (a large number of zero data appears in the data set). According to the idea of integrated learning, referring to the DeepFM [28] model (a highly efficient click rate prediction model of recommendation system), we integrate FM and LSTM in parallel, and the FM+LSTM model is constructed, which has the characteristics of automatic low-order feature combination, linear data connection, and sparse matrix friendliness. LSTM can solve the gradient explosion problem and has the characteristics of achieving high-order feature combination and nonlinear mapping. In addition, EEG data is obtained from sequential testing in a time series, which is very suitable for the prediction and classification of long-term and short-term memory models. Therefore, the proposed depth factorization machine model is suitable for two classification problems that determine eye behavior. The depth factorization machine model combines the advantages of FM and LSTM, and it is also applicable to two classification problems for judging the eye state. In the following section, we will introduce the FM model and the LSTM model briefly.

### 2.1. Introduction to FM

The factorization machine is a new model proposed by Rendle in 2010, which uses the idea of the implicit factor model and matrix decomposition for reference [29]. The problem of data sparsity can be overcome to some extent by using the idea of matrix decomposition to solve the optimal quadratic parameters. At present, many implicit factor models have been used for rating prediction in recommended fields, such as the common matrix decomposition, and the representative algorithm is singular value decomposition [30]. The main disadvantage of these algorithms is that they are only suitable for specific input data types, and the optimization algorithm is only proposed for the current task, which does not have generality. In different task scenarios, they cannot be directly migrated and extended horizontally. The factorization machine model is a general model, which can change the shortcomings of a traditional matrix decomposition algorithm. The FM only needs to change the form of the input eigenvector to simulate the common matrix decomposition model, while the traditional matrix decomposition model has to define model expression and optimization method separately for each specific task. Therefore, FM effectively avoids this kind of malpractice.

### 2.2. Introduction of LSTM

Because the artificial neural network can model the nonlinear process, it can solve a series of complex problems such as classification, clustering, dimensionality reduction, regression, and structural prediction. With the revolution of the computer industry, the exponential improvement of computer computing power, and the explosive growth of data in recent years, the deep-seated artificial neural network which needs a lot of computing power has been widely used. From the most classical perceptron, there are many kinds of neural network models. The typical models are the Convolutional Neural Network (CNN), recurrent neural network (RNN), and RNN variant LSTM. Different neural network models have different scenarios.

As mentioned above, the cyclic neural network or RNN is a kind of neural network for processing sequence data [31]. The network structure of LSTM was proposed by Hochreiter and Schmidhuber in 1997 [32], and then, this kind of network became very popular. Many people solved many practical problems based on the network structure of LSTM, and now, LSTM is still widely used. The recurrent neural network is a chain loop structure, and the network structure of LSTM is basically the same structure, but LSTM has a more complex structure in the network; therefore, it can deal with long-term dependence. LSTM has three gates to control, which are input gate, forgetting gate, and output gate. The input gate controls the input of the network, the forgetting gate controls the memory unit, and the output gate controls the output of the network. The most important one is the forgetting gate. The function of the forgetting gate is to decide which memories will be preserved and which memories will be forgotten. It is precisely because the function of forgetting gate “LSTM” has a long-term memory function. For a given task, the forgetting gate can be used to learn how many previous memories it can retain, which makes the network have the ability of learning autonomously without human interference.

**Figure 1:**
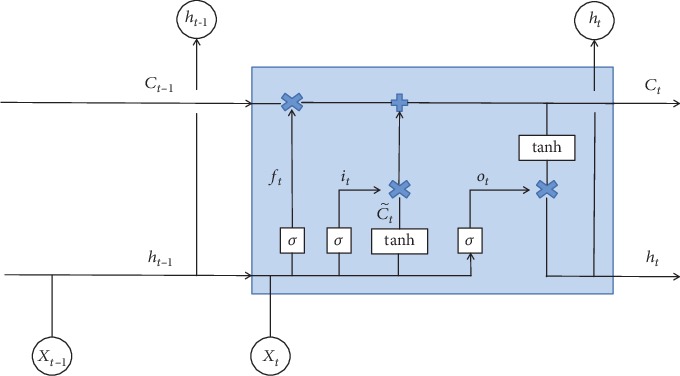
Internal structure of LSTM.

The following explains the network flow process of LSTM from the specific internal structure. The internal structure of the LSTM is shown in Figure 1. Next, we will explain their internal operation mode and how to represent the three gates mentioned above.

Input gate *i*_*t*_ controls how much information can flow into memory cells. Forgetting gate *f*_*t*_ controls how much information in the memory cells of the last moment can be accumulated into the memory cells of the current moment. Output gate *o*_*t*_ controls how much information in the memory cells of the current time can flow into the current hidden state *h*_*t*_.

*Step one*: use the forgetting gate to decide what information to discard from the cell state and calculate the attenuation coefficient. Read *h*_*t*−1_ and *x*_*t*_ and output a value between 0 and 1 to each number in cell loading *C*_*t*−1_. This determines what information we discard from the state of the cell. Since the output of sigmoid is 0 to 1, 1 indicates “full reservation” and 0 indicates “complete abandonment.”

*Step two*: update the information. First, the sigmoid layer is the “input gate layer,” which determines what value we will update. Then, the tanh layer creates a new candidate vector.

*Step three*: update the time of old cell status *C*_*t*−1_, which is updated to *C*_*t*_.

*Step four*: the output gate decides what value to output. The output at this time is calculated according to the *C*_*t*_ state of the third part.

### 2.3. Methodology and Contribution

The research content of this paper is to detect the eye state (open eyes, closed eyes) through EEG data. The purpose is to detect the eye state for a long time, so as to diagnose the fatigue and health status of individuals. In fact, this is a two classification problem. Specifically, in this paper, we extract feature data from EEG by wavelet transform technology and then build a FM+LSTM model to classify and predict the eye state. This model combines the linear interaction characteristics of FM and the nonlinear interaction characteristics of LSTM, which can greatly improve the classification efficiency. The main contributions of this paper can be summarized as follows:

1. The feature is extracted from EEG by wavelet transform:

- The first-order low-frequency signal features of EEG data are obtained by wavelet transform [33], and there are 14 EEG values
- The extracted data features are used as the basis for our classification and prediction

(2) Build a FM+LSTM model to classify and predict the eye state:

- This model is the first that we have established and applied to the study of eye behavior classification and prediction based on EEG
- The model is composed of FM and short-term memory neural network in a parallel form, which combines the advantages of both models with the characteristics of automatic low-order feature combination, linear data connection, and sparse matrix friendliness. LSTM can be used to solve the problem of gradient explosion and realize high-order feature combination and nonlinear mapping, which is suitable for the classification and prediction of eye behavior in this paper

(3) In this paper, the model is tested on the real data set, and through the comparison of the experimental results, it is found that the FM+LSTM model established in this paper has better classification and prediction efficiency than other classification models

## 3. EEG Signal-Based FM+LSTM Model

### 3.1. Theoretical Framework

**Figure 2:**
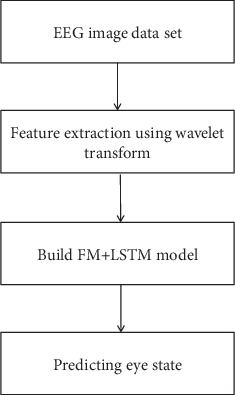
The framework of the proposed method.

In the experimental part of this paper, we will first introduce the theoretical framework of the experimental content, as shown in Figure 2.

### 3.2. Feature Extraction of EEG by Wavelet Transform

The EEG signal is a nonlinear and nonstationary random weak signal, with a relatively weak amplitude. Generally, the amplitude of the EEG signal is not more than 300 *μ*V. Continuous wavelet transform (CWT) [33–36] essentially convolutes the EEG signal with the translation dilation wavelet weight function with localization in a time and frequency domain, so as to decompose the signal into various components in different times or frequency. Let the signal *f*(*t*) of EEG be the square integrable function, which is recorded as *f*(*t*) ∈ *L*^2^(*R*). Convolute the EEG signal *f*(*t*) with the mother wavelet function, and call Equation (1) the continuous wavelet transform of the EEG signal *f*(*t*):

(1) $$\begin{array}{c} {\text{WT}}_{f}\left(a,\tau \right)=\langle f\left(t\right),\psi_{a,\tau }\left(t\right)\rangle =a^{-1/2}\int_{R}f\left(t\right)\bar{\psi \left(\frac{t-\tau }{a}\right)}dt. \end{array}$$

In the above formula, WT_*f*_(*a*, *τ*) is the wavelet coefficient of the EEG signal, scale *a* controls the expansion and contraction of wavelet function, and translation amount *τ* controls the translation of wavelet function. The scale corresponds to frequency (inverse ratio), and translation *τ* corresponds to time.

If the Fourier transform $\overset{⌢}{\psi }\left(w\right)$ of the parent wavelet function satisfies the constant resolution constraint

(2) $$\begin{array}{c} C_{\psi }=\int_{0}^{\infty }\frac{{\left|\psi \left(w\right)\right|}^{2}}{w}dw<\infty , \end{array}$$

then there is a reconstruction formula for the continuous wavelet transform of the EEG signal, and its expression is shown in

(3) $$\begin{array}{c} f\left(t\right)=\frac{1}{C_{\psi }}\int_{0}^{\infty }\frac{da}{a^{2}}\int_{R}{WT}_{x}\left(a,\tau \right)\frac{1}{\sqrt{a}}\psi \left(\frac{t-\tau }{a}\right)d\tau . \end{array}$$

In the above formula, *C*_*ψ*_ is the permissive condition of *ψ*(*t*) and WT_*x*_(*a*, *t*) is the wavelet transform coefficient. Since the wavelet base has two parameters of scale and displacement, the expansion of the wavelet base means that one-hour function is projected on the two-dimensional time-scale phase plane. And because of the characteristics of the basic body of wavelet, the function projection to wavelet transform is conducive to extract some features.

Because of the invariable window and the excellent characteristics of local analysis, the characteristics of the EEG signal in different frequencies can be analyzed by wavelet transform in different time scales.

### 3.3. FM+LSTM Model Construction Method

For a classification and prediction system, it is very important to learn the EEG feature combination behind the real eye state. Among the features extracted from EEG, low-order combined features (including one-dimensional and two-dimensional feature data) or high-order combined features (multidimensional feature data) may affect the final classification and prediction results. Considering the disadvantages of traditional classification methods, it includes the following:

1. The decision tree model easily leads to overfitting and ignores the correlation between data
2. The random forest model is prone to overfitting when the data noise is large
3. The logistic regression model cannot combine features and depends on artificial feature combination
4. The prediction process of the SVM model depends on the support vector in the training sample

We can see that the traditional classification algorithm cannot complete the classification task well, and therefore, we use the FM in this paper. The factorization machine model extracts the feature combination through the implicit variable inner product of each one-dimensional feature, which can extract the combined feature actively, keeps independent of the training sample but not rely on the support vector, and will not easily be affected by the noise data and appear the phenomenon of overfitting. However, in theory, FM can model higher-order feature combinations; in fact, only the second-order feature combination is used because of the complexity of calculation. In this paper, we solve the problem of high-order feature combination by multilayer neural networks which can learn the nonlinear complex relationship. We further use LSTM, which is a variation of RNN. LSTM contains the characteristics of a traditional neural network which is composed of the input layer, hidden layer, and output layer. LSTM also inherits all the advantages of RNN. It can adjust the parameters through back propagation. Moreover, LSTM can make the neurons in the hidden layer communicate with each other and establish the connection between the characteristic data. Not only that, the LSTM also solves the problem that the circulating neural network is prone to gradient explosion. Therefore, the LSTM contains the advantages of many neural networks, and its performance has been improved. And it also has a good performance in the classification problem.

Based on the advantages of the FM model and LSTM, we build the FM+LSTM model on the basis of the DeepFM [28] model to solve the classification problem. It effectively combines the advantages of FM and LSTM in feature learning: It can extract low-order combined features and high-order combined features at the same time. In the FM+LSTM model, we use a parallel approach to build our depth factorization machine model. Through the depth factorization machine model, on the one hand, FM can be used to extract the features from the first-order features and second-order features which are by the combination of the pair first-order features; on the other hand, LSTM can be used to extract features from the higher-order features which is formed by the input first-order features. Specifically, the characteristics of the FM+LSTM model include

1. being able to process sparse data
2. combining the FM model and the LSTM model, learning low-order feature combination and high-order feature combination at the same time

To sum up, we explained the principle of the FM+LSTM model and the advantages of the model. Next, we will show the mathematical principle of the model.

First, we use the FM [29]. The second-order expression of the algorithm is shown in

(4) $$\begin{array}{c} y\left(x\right)=w_{0}+\overset{n}{\underset{i=1}{\sum }}w_{i}x_{i}+\overset{n}{\underset{i=1}{\sum }}\overset{n}{\underset{j=i+1}{\sum }}w_{ij}x_{i}x_{j}, \end{array}$$

where *n* represents the feature dimension, *x*_*i*_ represents the feature value of the first feature, and *w*_*i*_ and *w*_*ij*_ are the coefficients of the primary term and the secondary term of the factorization machine model, respectively. A symmetric matrix *w* is composed of all the parameters *w*_*ij*_ of quadratic terms. At this time, the matrix can be decomposed into the following forms: *w* = *v*^*T*^*v*, where the *i*th column of *v* is the corresponding vector expression of the *i*th feature, and at this time, the coefficient of quadratic terms can be expressed as the inner product of two vectors, namely, *w*_*ij*_ = 〈*v*_*i*_, *v*_*j*_〉. In this case, the expression of the second-order model is as shown in

(5) $$\begin{array}{c} y_{\text{FM}}=w_{0}+\overset{n}{\underset{i=1}{\sum }}w_{i}x_{i}+\overset{n}{\underset{i=1}{\sum }}\overset{n}{\underset{j=i+1}{\sum }}\langle v_{i},v_{j}\rangle x_{i}x_{j}, \end{array}$$

where *v*_*i*_ is the vector expression of the *i*th feature *k*, dimension *k*, and <·, ·> is the vector dot product. At this time, the coefficients of the quadratic terms *x*_*h*_*x*_*i*_ and *x*_*i*_*x*_*j*_ are no longer independent. Specifically, the coefficients of *x*_*h*_*x*_*i*_ and *x*_*i*_*x*_*j*_ are <*x*_*h*_, *x*_*i*_> and <*x*_*i*_, *x*_*j*_>, which have the same term *v*_*i*_. Then, all the samples with nonzero feature combination of *x*_*i*_ can be used to learn *v*_*i*_.

In the LSTM model [32], the output is controlled by the forgetting gate, input gate, and output gate. The forgetting gate is used to determine what information is discarded from the cell state, and the attenuation coefficient is calculated as shown in formula (6). Read *h*_*t*−1_ and *x*_*t*_, and output a value between 0 and 1 to each number in cell loading *C*_*t*−1_. This determines what information we discard from the state of the cell. Since the output of sigmoid is 0 to 1, 1 indicates “full reservation” and 0 indicates “complete abandonment”:

(6) $$\begin{array}{c} f_{t}=\sigma \left(W_{f}\cdot \left[H_{t-1},x_{t}\right]+b_{f}\right). \end{array}$$

In the above formula, *W*_*f*_ is the weight value, *t* is the number of input data, *t* is the range of *t* = {0, 1, ⋯, *T*}, *x*_*t*_ is the *t*th input data, *H*_*t*−1_ is the output result of *t*‐1 neuron, *b*_*f*_ is the deviation value, and *σ*(·) is the activation function sigmoid, which is defined as shown in

(7) $$\begin{array}{c} f\left(x\right)=\frac{1}{1+e^{-x}}. \end{array}$$

Use the input gate to decide what value we are going to update. Then, the tanh layer creates a new candidate vector. The calculation equation is as follows:

(8) $$\begin{array}{c} i_{t}=\sigma \left(W_{i}\cdot \left[h_{t-1},x_{t}\right]+b_{i}\right),\\ {\tilde{C}}_{t}=\tanh\left(W_{c}\cdot \left[h_{t-1},x_{t}\right]+b_{c}\right). \end{array}$$

In the above formula, *σ*(·) is the activation function sigmoid, *W*_*i*_ and *W*_*o*_ are weight values, *b*_*i*_ and *b*_*o*_ are deviation values, and tanh(·) is the activation function. Its definition is as shown in

(9) $$\begin{array}{c} f\left(x\right)=\frac{1-e^{-2x}}{1+e^{-2x}}. \end{array}$$

When updating the old cell status, *C*_*t*‐1_ is updated to *C*_*t*_. The calculation formula is shown in

(10) $$\begin{array}{c} C_{t}=f_{t}*C_{t-1}+i_{t}*{\tilde{C}}_{t}. \end{array}$$

Use the output gate to decide what value to output. The output at this time is calculated according to the *C*_*t*_ state of the third part. The calculation process is shown in

(11) $$\begin{array}{c} O_{t}=\sigma \left(W_{o}\left[h_{t-1},x_{t}\right]+b_{o}\right),\\ H_{t}=O_{t}*\tanh\left(C_{t}\right). \end{array}$$

In the above formula, *σ*(·) is the activation function sigmoid, *W*_*o*_ is the weight value, and *b*_*o*_ is the deviation value.

The output results of each nerve unit can be obtained through the above steps. Finally, the output results of the neuron (the *t*th) can be obtained under the effect of output and transmission of different cycle units:

(12) $$\begin{array}{c} y_{\text{LSTM}}=\sigma \left(W_{t}\cdot H_{t}+b_{t}\right). \end{array}$$

In the above formula, *σ*(·) is the activation function sigmoid, *W*_*t*_ is the weight value, and *b*_*t*_ is the deviation value.

Finally, we combine the output of FM and LSTM to get the prediction results as shown in

(13) $$\begin{array}{c} y_{\text{prediction}}=\text{sigmoid}\left(y_{\text{FM}}+y_{\text{LSTM}}\right). \end{array}$$

We use logloss as the loss function of the depth factor decomposition machine model constructed in this paper, and then use gradient optimization algorithm to adjust the parameters, and finally get the optimal parameters.

**Figure 3:**
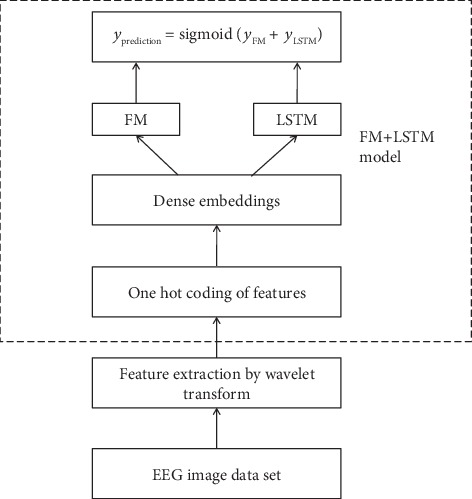
Overall flow chart of the FM+LSTM model.

We have introduced the mathematical principle of the depth factor decomposition machine model in detail. Next, we will show the overall flow chart of building the FM+LSTM model, as shown in Figure 3.

## 4. Experiment and Execution Results

### 4.1. Classified Forecast

In the task of classification and prediction, we compare the effect of the model used in this paper with the following benchmark models.

1. *Decision Tree (DT) Algorithm* [37]. It is a method of approaching the value of discrete function, which is a typical classification method. Firstly, the data is processed, the readable rules and decision trees are generated by using an induction algorithm, and then the new data is analyzed by using decision. In essence, the decision tree is a process of data classification through a series of rules.
2. *Random Forest (RF) Algorithm* [38]. Random forest is an algorithm that integrates multiple trees through the idea of integrated learning. Its basic unit is the decision tree, and its essence belongs to a big branch of machine learning—ensemble learning method.
3. *Logistic Regression (LR) Algorithm* [39]. It is used to deal with the regression problem when the dependent variable is classified variable. The common problem is the binomial or binomial distribution problem. It can also deal with the multiclassification problem. In fact, it belongs to a classification method.
4. *Support Vector Machines* [40]. SVM finds a hyperplane to divide the data into one class and other classes, which is a two-class classification model. The separation interval is the largest and different from the perceptron.

### 4.2. Prediction Scheme

In order to evaluate the prediction efficiency of the classification prediction task, we use common evaluation indicators: accuracy, precision, recall, and F1-measure. Specifically, the definitions of accuracy, precision, recall, and F1-measure are as follows [26]:

(14) $$\begin{array}{c} \text{Accuracy}=\frac{\text{TP}+\text{TN}}{\text{TP}+\text{TN}+\text{FN}+\text{FP}},\\ \text{Precision}=\frac{\text{TP}}{\text{TP}+\text{FP}},\\ \text{Recall}=\frac{\text{TP}}{\text{TP}+\text{FN}},\\ \text{F}1‐\text{Measure}=\frac{2\times \text{Recall}\times \text{Precision}}{\text{Recall}+\text{Precision}}. \end{array}$$

TP is the number of correctly recognized closed eyes, FP is the number of incorrectly recognized closed eyes, TN is the number of correctly recognized open eyes, and FN is the number of incorrectly recognized open eyes. The accuracy rate is the ratio of the number of samples correctly classified by the classifier to the total number of samples in a given test data set, the accuracy is the ratio of the number of correctly predicted samples in all predictions, the recall rate is the correctly predicted positive samples in all actual prediction proportion, and the F1-measure is the harmonic average of the exact value and the recall rate. These four evaluation schemes help us compare the efficiency of different classification models in the EEG data set for eye behavior prediction, and the bigger the results of the four evaluation methods, the closer the prediction results to the actual situation.

In this paper, the accuracy, precision, recall rate, and F1-measure are used to evaluate the prediction efficiency of different models. They are all classic evaluation methods of binary prediction which are suitable for the eye behavior (open and close eyes) classification prediction based on EEG data. Among them, accuracy is the ratio of the number of samples correctly classified by the classifier to the total number of samples in a given test data set, accuracy is the proportion of the number of samples correctly predicted as positive in all predictions, recall rate is the proportion of samples correctly predicted as positive in all actual predictions, and F1-measure is the harmonic average of the accuracy and recall rate. These four evaluation schemes help us to compare the efficiency of different classification models in EEG data set for eye behavior prediction, and the bigger the results of the four evaluation methods, the closer the prediction results to the actual situation.

### 4.3. Comparison of Classified Prediction Results

The following table is the classification prediction performance of the model used in this paper and other benchmark models on the EEG_EYE data set, and the specific results are shown in Table 1.

**Figure 4:**
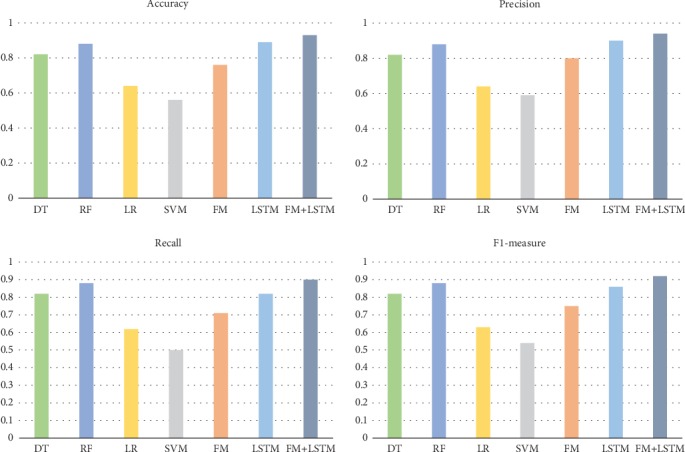
Histogram of prediction results of each classification model.

As shown in Table 1, we sorted out the classification and prediction results of various classifiers on the EEG data set. In order to compare the effects of various classifier models more intuitively, we drew a histogram as shown in Figure 4.

In the traditional classification prediction model, the logical regression model has the advantages of easy use and understanding but has the disadvantages of not being able to extract the combination features actively; the decision tree model has the advantages of not being sensitive to the missing value and not being able to process the data type and the conventional type attributes at the same time but has the disadvantages of low antioverfitting ability and being easily disturbed by the noise data; the random forest model has the advantages of strong antioverfitting ability and balance error, but it is also vulnerable to the interference of noise data; the support vector machine model has the advantages of finding the global minimum value through the convex optimization method, but it has the disadvantages of relying too much on support vector and processing large-scale training data; the factorizer model has the advantages of high learning efficiency and processing large-scale sparse data, but it has the disadvantages of only extracting the second-order feature combination; the LSTM model has the advantages of processing large-scale sparse data and solving the problem of long sequence dependence, but it can only extract the high-order feature combination and cannot combine the high-order feature with the low-order feature.

Specifically, by combining the classification results of Table 1 and Figure 2, we can get the following conclusions:

1. In the test set of EEG_EYE data, the overall classification effect of the factorization machine model is better than that of the logistic regression model and the support vector machine model; this shows that the factorization model has the characteristics of extracting the combined features actively and the characteristics of keeping independent of training samples, which makes it better than the above logistic regression model and support the vector machine model in classification and prediction results
2. In the test set of EEG_EYE data, the overall classification effect of LSTM is better than that of the decision tree model and random forest model, which shows that the LSTM has no serious overfitting linearity due to the noise interference in the test set, and therefore, the classification and prediction effect is better than the other two classification models
3. In the test set of EEG_EYE data, the model of FM+LSTM is better than other models in classification and prediction. This is because the model can extract low-order combined features and high-order combined features at the same time, which makes up for the disadvantage that LSTM can only extract high-order feature combinations

In summary, the FM+LSTM model established by us can not only extract the combined features actively but also reduce the noise interference on the simulation. In addition, it can extract both low-order combined features and high-order combined features; therefore, its performance is better than other models.

## 5. Future Work

In this paper, we established a FM+LSTM model to predict the eye behavior (open or close eyes) of the user by EEG data, so as to infer whether the user is in a fatigue state. Simulation results show that the proposed method achieved good classification and prediction results. The direct significance of this work is that we can judge whether the user is in the fatigue state through the EEG data. Furthermore, the fatigue state is also an important feature of the user in the recommendation system, which can affect the user's preference and evaluation of the items. Therefore, in the future work, first, we plan to further study the user's preference and evaluation of the different items based on the proposed research achievement of the eye's fatigue state which is an important feature factor related to the context, so as to improve the efficiency of recommendation. Second, we plan to use the attention mechanism to distinguish the influence of various features on eye behavior, so as to enhance the interpretability of the classification prediction result. In addition, based on the research results of this paper, we plan to further use the graph neural network model to study the complex interaction between different features in a more flexible and clearer way, so as to obtain more efficient and more accurate recommendation results.

## 6. Conclusions

The purpose of this paper is to predict the eye state (open and closed eyes) by EEG data, which is a typical dichotomous problem. The significance of this study is to diagnose the fatigue and the health of the eyes by detecting the eye state for a long time. And on the basis of this research, we can also infer the fatigue degree of the human body by monitoring the eye state of the human body for a long time, so that we can help in user behavior recommendation based on this inferential. Firstly, we use wavelet transform technology to extract the features of EEG, and then, we use a classifier to classify and predict. Because the traditional binary classification has many shortcomings in some aspects, and its performance is poor, we use the FM algorithm which is widely used in the click rate prediction of the recommendation system and binary classification algorithm. In addition, we also use LSTM, which is a deep network model with good generalization ability and nonlinear mapping ability. According to the idea of integrated learning, referring to the DeepFM [28] model (a highly efficient prediction model of click through rate of the recommendation system), we integrate FM and LSTM in parallel and build the FM+LSTM model, which combines the advantages of FM and LSTM and is suitable for the two classification problems of the eye state in this paper. And its performance in real data set is better than other classifier models. Through this research, we can also infer the fatigue degree of the human body by monitoring the eye state of the human body for a long time, so that we can help in user behavior recommendation based on this inference, which will be the focus of our next research. In this paper, the FM+LSTM model is established to solve the problem of eye behavior classification and prediction. The conclusion obtained in this paper will be an important factor in the determination of user preferences in the recommendation system, which will be used in the analysis of interactive features by the graph neural network in the future work.

## Figures and Tables

**Table 1:** Classification prediction results of various methods.

Algorithm/model	Accuracy	Precision	Recall	F1-measure
DT	0.82	0.82	0.82	0.82
RF	0.88	0.88	0.88	0.88
LR	0.64	0.64	0.62	0.63
SVM	0.56	0.59	0.50	0.54
FM	0.76	0.80	0.71	0.75
LSTM	0.89	0.90	0.82	0.86
FM+LSTM	0.93	0.94	0.90	0.92

## Data Availability

The data set used in this experiment is collected by Oliver rothler from Germany, Stuttgart and Baden Wurttemberg state cooperative University (DHBW). The data set is composed of EEG value and a value indicating eye state, which belongs to the field of life medicine. All the data are the result of continuous EEG measurement with Emotiv. The measurement duration is 117 seconds. During the EEG measurement, the eye state is detected by the camera, and then manually added to the file after analyzing the video frame. "1" means that the eyes are closed, and "0" means that the eyes are open. For more information about the data, please visit: http://archive.ics.uci.edu/ml/datasets/EEG+eye+state.
